# Incorporation of high fructose corn syrup with different fructose levels into biscuit: An assessment of physicochemical and textural properties

**DOI:** 10.1002/fsn3.2452

**Published:** 2021-08-01

**Authors:** Arash Ershadi, Mohammad Hossein Azizi, Leila Najafian

**Affiliations:** ^1^ Department of Food Science and Technology Sari Branch Islamic Azad University Sari Iran; ^2^ Department of Food Science and Technology Faculty of Agriculture Tarbiat Modares University Tehran Iran

**Keywords:** acrylamide, biscuit, HFCS, HMF, physicochemical properties

## Abstract

This study examined the effects of different concentrations of high‐fructose corn syrup (HFCS, 28%, 44%, 55%) used in biscuit formulation on the hydroxymethyl furfural (HMF) acrylamide content, and textural properties were investigated and compared with invert sugar and sucrose‐incorporated samples. No significant difference in the chemical composition (moisture, fat, protein, and ash) among different samples was noted based on the results. The highest L* was associated with a control sample containing sugar and invert sugar, although an increase in F55 content decreased the L* value significantly (*p* < .05). The highest hardness value was correlated with control samples (6.5 N), although the sample with 12.5% F42 and 25% F55 demonstrated lower hardness 6.27 N, and the lowest hardness value (3.97 N) was related to the sample containing 12.5% F42 and 25% F28. The amounts of water activity of all samples were in the range of 0.22 to 0.29, with the highest amount related to the control sample. The *SEM* images showed a uniform surface with several holes for all the biscuits. The highest and lowest (HMF) levels were related to the samples containing 25% F55 (46.04) and 12.5% F42 with 2.36 ppm. The control sample with the acrylamide amount of 28.50 ppb and the sample containing 12.5% F42 and 25% F55 with the acrylamide amount of 27.33 ppb showed the highest acrylamide content.

## Graphical abstract

This study examined the effects of different concentrations of high fructose corn syrup (HFCS, 28, 44, 55%) used in biscuit formulation on the Hydroxymethyl furfural (HMF) and acrylamide content and textural properties were investigated and compared with invert sugar and sucrose incorporated samples.

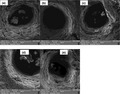

## 1 INTRODUCTION

The application of high‐fructose corn syrup (HFCS) has gradually been enhanced in the United States, corresponding to 40% of total caloric sweeteners. A 1,000% increase in HFCS use was reported from 1970 to 1990, accompanied by decreased sucrose use (Goncalves et al., 2019). HFCS is usually used in the formulation of caloric beverages such as colas and soft drinks (Bocarsly et al., 2010) and may be applied as an ingredient in different products, including bakery goods, bread, cereals, desserts, fruit juice, jellies, and jams, and canned foods (Zargaraan et al., 2016) (Berger et al., 2018) (Bocarsly et al., 2010).

The biscuit formula is composed of wheat flour, sugar water, fat, and salt. After mixing these ingredients and adding other ingredients, including skimmed milk, baking powder, sodium sulfite, emulsifier, and biscuit dough with useful gluten network forms. The quality of a biscuit is dependent on the quantity and quality of its ingredients (Mamat et al., 2010).

Among the heated bakery products, including biscuit and bread, acrylamide and hydroxymethyl furfural (HMF) levels have been investigated using different studies. The neurotoxic, mutagenic, and carcinogenic effects of acrylamide have been proved on human and animal cells (Shipp et al., 2006). Genotoxic and mutagenic impacts of HMF on human cells, as well as bacteria, are reported. HMF can promote liver and colon cancers in animals such as mice and rats (Glatt & Sommer, 2006; Monien et al., 2012). However, more robust and clear evidence for genotoxic and carcinogenic impacts of HMF is needed (Capuano & Fogliano, 2011).

Few studies have been performed regarding the effects of HFCS with different fructose levels on biscuit properties. In this study, three HFCS with different fructose levels (28%, 44%, and 55%) were used in biscuit formulations, and their effects on sensorial, physicochemical (especially HMF and acrylamide), textural, and microbial products were investigated.

## 2 MATERIAL AND METHODS

### 2.1 Materials

Three HFCSs with different fructose levels, including 28%, 44%, and 55%, were obtained from Zar Co. Other chemical reagents, all in analytical grade, were obtained from Merck and Sigma Aldrich Co. Oil and sucrose were purchased from a local market in Tehran, Iran.

### 2.2 Sample preparation

Five different biscuit formulation trials were used in this study (Table 1). Preparation of biscuit dough was performed based on the following steps: (a) mixing solid oil, flavor, and lecithin for 5 min; (b) adding milk powder, egg powder, sucrose, salt, and sodium bicarbonate and mixing for 5 min; (c) adding HFCS or invert sugar to the mixture and mix for 2 min; (d) adding water and mixing for 4 min; (e) adding malt followed by 5 min mixing; and (f) adding wholemeal flour and mixing for 15 min. Mixing in all steps was done using a mixer equipped with a flat beater (Kitchen Aid). The prepared dough was then sheeted in a tray (5 mm thickness) and then cut using a round cutter (50 mm internal diameter). Finally, the cut dough was baked in an oven at 160°C for 20 min. Intact biscuits were used for colorimetric analysis and textural and sensory evaluations, and freshly crushed biscuit (using a mortar) was used for other analyses. The prepared samples were S(87.5)‐I(12.5), 87.5% sugar and 12.5% invert sugar; S(87.5)‐F42(12.5), 87.5% sugar and 12.5% HFCS 42; S(62.5)‐F42(12.5)‐F55(25), 62.5% sugar and 12.5% HFCS 42 and 25% HFCS 55; S(62.5)‐F42(12.5)‐F42(25), 62.5% sugar and 37.5% HFCS 42; and S(62.5)‐F42(12.5)‐F28(25), 62.5% sugar and 25% HFCS 28 and 12.5% HFCS 42.

**TABLE 1:** Sweetener ingredients of biscuits

	Sugar (%)	Invert sugar (%)	HFCS 28 (%)	HFCS 42 (%)	HFCS 55 (%)
S(87.5)–I(12.5)	87.5	12.5	0	0	0
S(87.5)–F42(12.5)	87.5	0	0	12.5	0
S(62.5)–F42(12.5)–F55(25)	62.5	0	0	12.5	25
S(62.5)–F42(12.5)–F42(25)	62.5	0	0	37.5	0
S(62.5)–F42(12.5)–F28(25)	62.5	0	25	12.5	0

### 2.3 Chemical composition

The chemical composition (moisture content by the oven method at 105°C, protein content by the Kjeldahl method, fat content by Soxhlet method, and ash content by the oven method at 600°C) of biscuit samples was estimated based on the standard analysis methods (Pasqualone et al., 2015).

### 2.4 Color measurement

Colorimetric analysis of the samples' outer surface was performed based on the proposed method by Hosseini et al. (2019). A Canon digital camera (A540, 6 megapixels) was located on the top of a wooden box with 50 cm length, 50 cm width, and 60 cm height to capture images. The distance between the camera lens and the samples in the box was adjusted to 25 cm. The box was lightened with a natural daylight source (6,500 K). Different color parameters, including lightness (L*), blueness‐yellowness (b*), and redness‐greenness (a*), were calculated using Adobe Photoshop^®^ CS6 from the taken images.

### 2.5 Texture measurement

The hardness of the fresh, intact biscuit was determined using a three‐point bend test. A texture analyzer (TAXT2) with the three‐point bending and 5 kg load cell and 40 mm span between the supports was used for the test. The test was performed with a pretest speed of 1.0 mm/s, a test speed of 3.0 mm/s, and a posttest speed of 10.0 mm/s, with a 10 mm distance and 500 PPS data acquisition rate. The maximum recorded force was considered as the hardness of samples. The test was repeated three times, and the average hardness and the distance to break were reported (Mamat et al., 2010).

### 2.6 Water activity measurement

The relative humidity (RH) of the air, equilibrated with the biscuits placed in a sealed container (22 ± 1°C), was reported as water activity (aw). A water activity meter (Aqua Lab Series 3) was used for this evaluation (Laguna et al., 2014).

### 2.7 Fourier transform infrared spectroscopy

The FT‐IR spectra of biscuit powders were recorded in the wavenumber range of 4,000–400 cm−^1^ by the Avatar 370 spectrometer (Thermo Nicolet Corp.). Initially, biscuit samples were completely powdered and mixed with KBr (Gahruie et al., 2019) and finally pressed to create a tablet.

### 2.8 *SEM* measurement

The Vega3 electron microscope (*SEM*) (TESCAN) was used to evaluate the biscuit samples' surface microstructure. In this regard, the small parts of the dried biscuits were attached to aluminum stubs by double‐sided adhesive tape. Then, a thin layer of gold was coated over the samples using Desk Sputter Coater DSR1, Nanostructural Coating Co. The accelerating voltage and temperature used in the test were 10 kV and 25°C, respectively (Gahruie et al., 2020).

### 2.9 Hydroxymethylfurfural measurement

To measure HMF in samples, 2 g of crushed biscuit was poured into a Greiner tube, and 3 ml of Mili‐Q water was added to it, vortexed for 1 min, followed by incubation 50°C for 1 hr. After centrifuging at 3,000 rpm for 10 min, 1,500 µl of the separated supernatant was removed and centrifuged for 15 min at 5,000 × *g*. The supernatant was collected and filtered through a 0.22‐µm filter and evaluated by an HPLC system. The HPLC system had a UV detector (248 nm) and a Polaris 5 C18‐A column (150 × 4.6 mm, 5 µm) to determine the amount of HMF. The mobile phase was water: acetonitrile (95:5) with a 1 ml/min flow rate and a typical run time of 20 min per sample. The analytical method had a limit of detection of 1 mg/kg sample powder. Measurements were carried out in duplicate. A standard method by a calibration curve was used to quantify HMF (Nguyen et al., 2016).

### 2.10 Acrylamide measurement

The amount of acrylamide was determined based on Wang et al. (2017). In brief, 1.0 g of completely dried biscuit and 5 ml methanol were mixed in a tube and centrifuged at 10,000 × *g* at 4°C (10 min). The supernatant was then removed, and 0.1 ml Carrez I and 0.1 ml Carrez II solutions were added to precipitate and centrifuged at 5,000 × *g* at 10°C (10 min). Then methanol was added to the supernatant until reaching 5 ml volume. Then 1 ml of the solution was evaporated by nitrogen purging at 40°C until reaching dryness ultimately and 1 ml distilled water was added to the residue.

A Thermo carbon column was conditioned with methanol (5 ml) and water (5 ml) sequentially for solid‐phase extraction cleanup. In the next step, 1 ml of the extract was injected into the solid‐phase extraction column to pass through the sorbent material. Elution of the acrylamide presented in the column was performed using methanol (5 ml), and then the elute was collected and evaporated with a nitrogen stream at 40°C. One milliliter of methanol was added to the residue until dissolving and then filtered through a 0.22‐µm polyvinylidene fluoride filter.

HPLC with a UV‐VIS detector (Shimadzu, Japan) was used to determine the amount of acrylamide. A Hypersil ODS‐C18 column (250 mm × 4.6 mm, 5 µm, Thermo Scientific) was used at 40°C for this purpose. The injection volume was 20 µl. The mobile phase was water: acetonitrile (95:5) with a flow rate of 0.6 ml/min. Acrylamide was detected at 210 nm, and its amount was determined using a calibration curve constructed in the concentration range of 0–10 µg/ml. CLASS‐VP Shimadzu automated software was used for data collection and manipulation.

### 2.11 Statistical analysis

All results were reported based on three replications. One‐way analysis of variance (ANOVA) at a significance level of 5% was used for statistical analysis of data, and the significant differences between the values were determined by Duncan's multiple range tests using SAS^®^ (ver. 9.1, SAS Institute Inc.).

## 3 RESULTS AND DISCUSSION

### 3.1 Chemical composition

Table 2 shows the effect of the fructose/glucose ratio on different chemical properties of samples. It can be observed that the incorporation of different sweeteners did not change the moisture, protein, fat, and ash contents of different biscuit samples significantly. These results were due to moisture, protein, fat, and ash contents related to basic formulation; other ingredients such as solid oil, flavor, and lecithin, milk powder, egg powder, and sweetener had similar moisture, protein, and protein, fat, and ash composition. The ranges of moisture content, protein content, fat, and ash were 2.07%–2.23%, 7.69%–7.79%, 14.35%–14.41%, and 0.96%–1.05%, respectively. According to Hooda and Jood (2005), the protein content, moisture content, and ash content of biscuit were 9%–10%, 3%–4%, and 1%–2%, respectively, which is almost similar to our results. In another work, biscuit's moisture content was 2%–4% Laguna et al. (2014). Akoja and Coker (2018) evaluated the effects of okra powder on the properties of wheat four biscuits. They reported that moisture content, ash, fat, and protein content of samples were 8%–10%, 1%–4%, 17%–20%, and 10%–21%, respectively.

**TABLE 2:** Chemical composition of samples

	Moisture content (%)	Ash (%)	Fat (%)	Protein (%)
S(87.5)–I(12.5)	2.23 ± 0.15A	1.05 ± 0.07A	14.35 ± 0.83A	7.79 ± 0.16A
S(87.5)–F42(12.5)	2.23 ± 0.31A	0.96 ± 0.06A	14.41 ± 0.84A	7.74 ± 0.30A
S(62.5)–F42(12.5)–F55(25)	2.07 ± 0.14A	1.01 ± 0.10A	14.41 ± 0.83A	7.69 ± 0.26A
S(62.5)–F42(12.5)–F42(25)	2.20 ± 0.21A	1.02 ± 0.06A	14.41 ± 0.85A	7.72 ± 0.15A
S(62.5)–F42(12.5)–F28(25)	2.20 ± 0.33A	1.01 ± 0.16A	14.35 ± 0.91A	7.74 ± 0.18A

Note

Data represent the mean ± standard deviation of three independent batches. Different uppercase letters in each column indicate significant differences (*p* < .05).

### 3.2 Color

**FIGURE 1:**
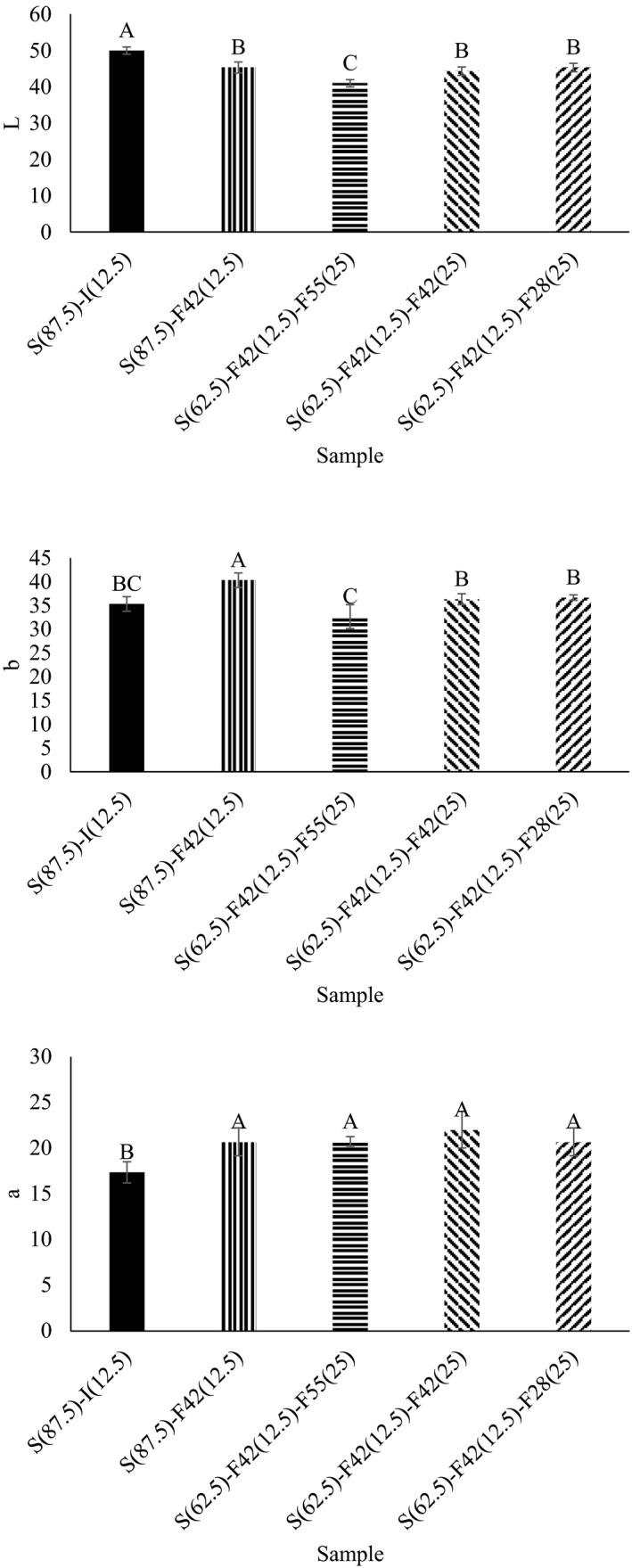
Color properties of samples

The samples incorporated with different sweeteners showed different colors (Figure 1). The control sample, which was incorporated with sugar and invert sugar, showed the highest lightness and the lowest a* value, although the lowest lightness was related to the sample containing 12.5% F42 and 25% F55, and the highest a* value belonged to all other samples containing fructose. These changes in the color are related to some interaction between sugar and protein in samples during cooking. Some of these reactions, such as Millard and caramelization, are important during cooking processing. The highest and lowest b* values were attributed to the biscuit containing 12.5% F42 and the biscuit containing 12.5% F42 and 25% F55, respectively. Pasqualone et al. (2015) reported that the biscuit's lightness was around 44 to 52. The evolution of the color could also be related to the browning associated with an advanced stage of Maillard reaction and caramelization (Mesías et al., 2016). Díaz et al. (2019) evaluated the effects of Jerusalem artichoke tuber flour on the color properties of biscuits. They reported that the L, a, and b values of the sample were 47–73, 4–8, and 14–31, respectively. Bolek (2020) evaluated the effects of olive stone powder on the color properties of biscuits. They reported L, a, and b of samples were 41–68, 11–15, and 18–34, respectively.

### 3.3 Texture

**FIGURE 2:**
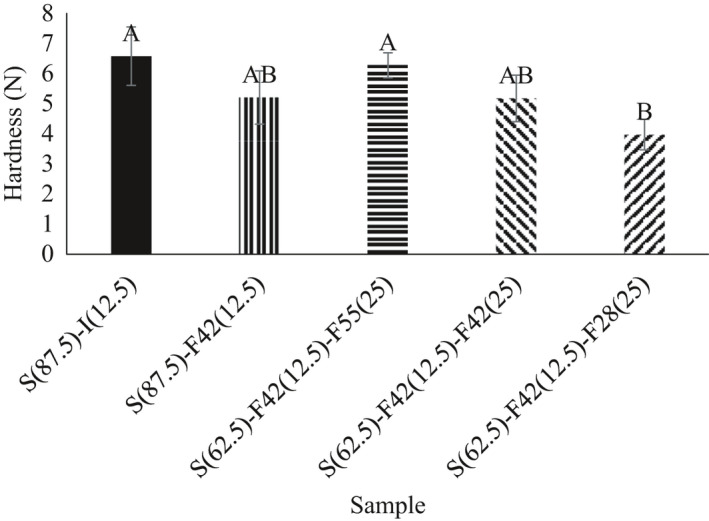
Hardness of samples

Figure 2 represents the hardness value of different biscuits containing different fructose/glucose ratios. Samples with different sweeteners had different hardness. The highest hardness was related to the control sample incorporated with sugar and invert sugar (6.57 N) and the sample containing 12.5% F42 and 25% F55 (6.27 N). The sample containing 12.5% F42 and 25% F28 with a value of 3.97 N had the lowest hardness. The maximum force recorded during fracturing biscuit samples was recorded as hardness value. Sugar replacement caused a significant reduction (*p* < .05) in the hardness of samples. The type of replacer and its concentration are the factors that determine the magnitude of hardness. Based on these results, higher glucose content in the formulation caused a lower hardness value. Díaz et al. (2019) evaluated the effects of Jerusalem artichoke tuber flour on the texture properties of biscuits. They reported that the hardness of the sample was between 7 and 41 N. Bolek (2020) evaluated the effects of olive stone powder on the texture properties of biscuits. They reported hardness of samples was between 29 and 41 N.

### 3.4 Water activity

**FIGURE 3:**
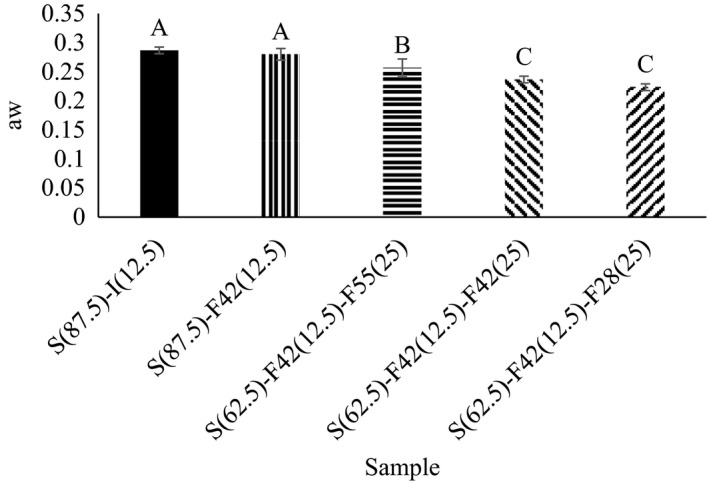
The water activity of samples

In biscuits and other brittle and dry foods, mechanical signatures and crispness are influenced by water activity, moisture content, and water distribution. There is a sigmoid relationship between water activity and content with crispness (Blanco Canalis et al., 2017; Laguna et al., 2014). Water activity measurement was performed to determine whether the sensory appreciation of crispness was influenced by water activity and moisture content or not. Based on Figure 3, a significant difference (*p* < .05) between different biscuit samples' water activity and different glucose/fructose ratios. The highest water activity was related to the control sample (0.29) containing sugar and invert sugar and the sample containing 12.5% F42 (0.28). Samples containing 37.5% F42 with 0.24 and sample containing 12.5% F42 and 25% F28 showed the lowest water activity values (0.24 and 0.22, respectively). This phenomenon is due to the high tendency of sugar (sucrose) to interact with water against glucose and fructose. There are differently reports on the effects of sugar composition on the water activity of systems. Also, the water activity of fructose and glucose solution is similar. The water activity of biscuit samples containing inulin and HPMC was reported to range from 0.12 to 0.25 (Laguna et al. (2014).

### 3.5 FTIR

**FIGURE 4:**
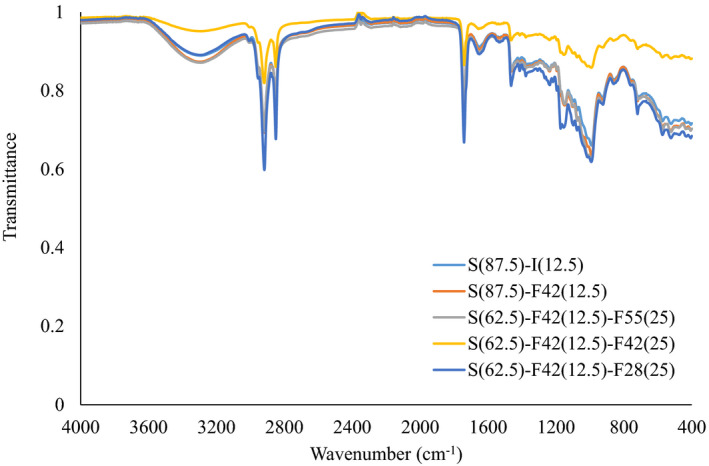
Fourier transform infrared spectroscopy of samples

Figure 4 represents the FT‐IR spectra of biscuits with different formulations. The FT‐IR spectrum of a sample shows the constituent functional groups of it. The typical peaks that occur in the range of 3,328–3,284 cm^−1^ indicate O–H bond stretching. The presence of tightly bound water in the biscuit samples caused a vibrational peak at 1,459–1,456 cm^‐1^. The peaks in the range of 3,328–2,853 cm^−1^ and 1,744–1,746 cm^−1^ were attributed to asymmetric stretching of the C‐H band and carbonyl stretch peaks. The peak related to amide I was observed in the range of 1,653–1,648 cm−1 region, and the peaks related to C = O bond stretching and aliphatic C = N stretching have appeared in the range of 1,158–1,020 cm^−1^. Adebiyi et al. (2016) reported similar results for the FT‐IR spectra of biscuit samples.

### 3.6 Microstructure properties

**FIGURE 5:**
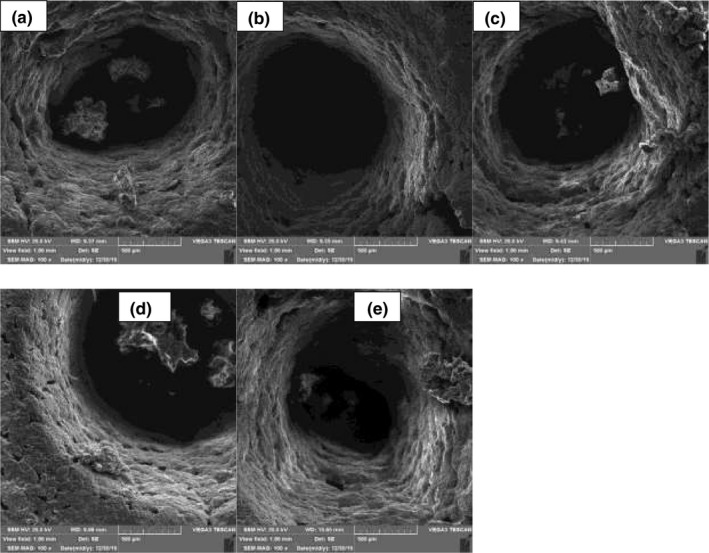
*SEM* micrograph of samples

The images of the microstructure of biscuit samples observed by the *SEM* are shown in Figure 5. As can be seen, all samples had a uniform surface with some holes on them. The microstructure of a biscuit can be described as a complex matrix composed of aggregations of protein, sugar, lipid, and damaged or undamaged starch granules Chevallier et al. (2000). The sugar type and concentrations are the affecting factors on starch gelatinization in biscuit samples (Filipčev et al., 2011; Krystyjan et al., 2015; Ratnawati et al., 2020).

### 3.7 HMF content

Based on Table 3, different glucose/fructose ratios resulted in different amounts of HMF in biscuit samples. The sample's HMF content containing 25% F55 was 46.04 ppm, which showed the highest value, and the samples incorporated with 37.5% F42, 12.5% F42, and 25% F28 had the lowest amounts of HMF (2.31 ppm and 4.05 ppm, respectively). The presence of invert sugar in the control and higher fructose in the sample containing F55 are the main reasons for higher HMF. Caramelization and a specific amino acid route are the parameters involved in the production of HMF (Nguyen et al., 2016). Nguyen et al. (2016) studied the impact of the sugar type on the amount of hydroxymethyl furfural in biscuits with different formulations and reported similar results. They declared that the amino acids present in the samples could increase the HMF production from glucose in the biscuit, although the formation of hydroxymethyl furfural from fructose (as the glucose substrate) was not affected by amino acids (LEE & NAGY, 1990). Mesías et al. (2016) evaluated the effects of chia flour on the properties such as HMF. They reported that the HMF content of samples was between 20 and 71 mg/kg. Values of HMF were included within the range of 3.1 to 182.5 mg/kg displayed in commercial biscuits marketed in Spain (Delgado‐Andrade et al., 2009).

**TABLE 3:** Hydroxymethyl furfural content and acrylamide content of samples

	HMF content (ppm)	Acrylamide content (ppb)
S(87.5)–I(12.5)	33.55 ± 2.31B	28.50 ± 0.45A
S(87.5)–F42(12.5)	2.36 ± 0.34C	23.51 ± 1.12B
S(62.5)–F42(12.5)–F55(25)	46.04 ± 1.69A	27.23 ± 1.39A
S(62.5)–F42(12.5)–F42(25)	2.31 ± 1.31C	24.74 ± 0.78B
S(62.5)–F42(12.5)–F28(25)	4.05 ± 0.98C	23.01 ± 1.34B

Note

Data represent the mean ± standard deviation of three independent batches. Different uppercase letters in each column indicate significant differences (*p* < .05).

### 3.8 Acrylamide content

As presented in Table 3, different glucose/fructose ratios in biscuit formulation resulted in different acrylamide contents in samples. The highest amounts were related to the control sample containing sugar and invert sugar with the value of 28.50 ppb and the sample containing 12.5% F42 and 25% F55 with 27.33 ppb, respectively. Other samples showed no significant differences (*p* < .05) in the amounts of acrylamide. The primary mechanism of production of acrylamide is related to the fructose content, and acrylamide content significantly increased with increasing fructose content. In a study on biscuits by Nguyen et al. (2016), it was revealed that in all the four biscuit types with different formulations, the production of acrylamide was mainly related to fructose. Similar results were also reported by Robert et al. (2005). Before the Maillard reaction, melting and other physical changes take place at a low‐moisture content medium. Because of the lower melting point of fructose than glucose, this sugar causes higher acrylamide production (De Vleeschouwer et al., 2009). Some studies have proved that the limiting factor in acrylamide production in yeast‐leavened wheat bread and heated wheat flour was free asparagine (Europe, 2015; Muttucumaru et al., 2006; Surdyk et al., 2004). Mesías et al. (2016) evaluated the effects of chia flour on the properties such as acrylamide. They reported that the acrylamide content of samples was between 151 and 1,187 mg/kg.

## 4 CONCLUSION

This study aimed to evaluate the effects of different amounts of high fructose corn syrup with different fructose levels on biscuit formulation compared with inverted sugar and sucrose. The results showed no significant differences among moisture, fat, protein, and ash content of different samples. The control sample had the highest and the sample containing F55 had the lowest L*. With the addition of F28, the hardness of samples significantly decreased. The water activity of all samples was in the range of 0.22–0.29. The FT‐IR spectra of all samples showed similar peaks with variations in their intensity. The *SEM* images showed a uniform surface with several holes on the biscuit surface. The highest HMF amount was related to the sample containing 25% F55 (46.04 ppm). The control sample with an acrylamide amount of 28.50 ppb and the sample containing 12.5% F42 and 25% F55 with an acrylamide amount of 27.33 ppb showed the highest acrylamide content. Finally, the results showed that the sample containing F42 was the best sample for replacing sucrose with HFCS.
